# Recent advances and future directions for uterine diseases diagnosis, pathogenesis, and management in dairy cows

**DOI:** 10.1590/1984-3143-AR2020-0063

**Published:** 2020-08-11

**Authors:** Fabio Soares de Lima

**Affiliations:** 1 Department of Population Health and Reproduction, School of Veterinary Medicine, University of California, Davis, CA, USA.

**Keywords:** metritis, endometritis, alternative therapies, prevention

## Abstract

Researchers, veterinarians, and farmers' pursuit of a consistent diagnosis, treatment, and prevention of uterine diseases remains challenging. The diagnosis and treatment of metritis is inconsistent, a concerning situation when considered the global threat of antimicrobial resistance dissemination. Endometritis is an insidious disease absent on routine health programs in many dairy farms and from pharmaceutical therapeutics arsenal in places like the US market. Conversely, a multitude of studies advanced the understanding of how uterine diseases compromise oocyte, follicle, and embryo development, and the uterine environment having long-lasting effects on fertility. The field of uterine disease microbiome also experienced tremendous progress and created opportunities for the development of novel preventives to improve the management of uterine diseases. Activity monitors, biomarkers, genomic selection, and machine learning predictive models are other innovative developments that have been explored in recent years to help mitigate the negative impacts of uterine diseases. Albeit novel tools such as vaccines for metritis, immune modulators, probiotics, genomic selection, and selective antimicrobial therapy are promising, further research is warranted to implement these technologies in a systematic and cost-effective manner.

## Introduction

Despite the undeniable detrimental impact of uterine diseases on fertility outcomes, culling risks, lactation performance, and sustainability of dairy operations (Bicalho et al., 2016; Lima et al., 2013, 2014, 2019), its diagnosis, treatment, and prevention remain inconsistent (Espadamala et al., 2018). A recent survey from 45 farms in California revealed that there is a significant disparity on which criteria are used to diagnosis and treat metritis (Espadamala et al., 2018), a concerning situation when considered the global threat of antimicrobial resistance dissemination and World Health Organization guideline on the use of medically important antibiotics (WHO, 2017). The situation of endometritis is even more daring with many farms performing no diagnosis and complete absence of efficacious therapy in places such as the US market (Haimerl et al., 2018). On the other hand, a myriad of research advances shed light on how uterine diseases may disrupt endocrine signaling, oocyte, follicle and embryo development, and the uterine environment (Bromfield et al., 2015; Horlock et al., 2020; Moore et al., 2019; Piersanti et al., 2019; Ribeiro, 2018). The understanding of uterine diseases microbiome also had tremendous progress and created opportunities for the development of novel preventives to improve the management of uterine diseases (Bicalho et al., 2012; Freick et al., 2017; Galvão et al., 2019a; Jeon et al., 2018; Machado et al., 2014; Sheldon et al., 2010). Indeed, in the last decade, over a dozen of non-antimicrobial therapeutics to prevent and treat metritis and endometritis has been investigated with encouraging results (Ahmadi et al., 2019; Ametaj et al., 2014; ATLAS Collaboration, 2017; Brick et al., 2012; Daetz et al., 2016; Escandon et al., 2020; Genis et al., 2018; Machado et al., 2012; Pinedo et al., 2015). Genome-enable prediction for health traits (McNeel et al., 2017; Lopes et al., 2020), activity monitors (Barragan et al., 2018; Stangaferro et al., 2016), biomarkers (Barragan et al., 2018, 2019; Dervishi et al., 2018; Wisnieski et al., 2019; Zhang et al., 2017), immune cells profile (Pomeroy et al., 2017), machine learning predictive models (Bogado Pascottini et al., 2020) are others innovative tools that have been explored in the recent years to help mitigate negative impacts of uterine diseases. The objective of this manuscript is to summarize recent advances and future directions for uterine disease diagnosis, pathogenesis, and management.

### Diagnosing uterine diseases: current status

**Figure 1:**
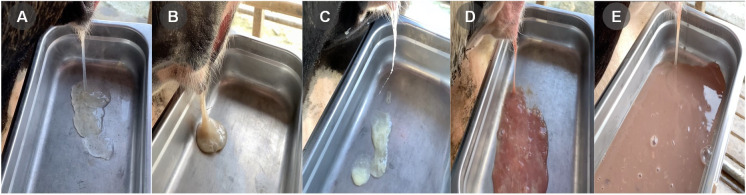
Vaginal discharge scoring system for postpartum dairy cows. (A) Clear discharge; (B) Cloudy discharge with speck of pus; (C) Purulent discharge; (D) Bloody purulent discharge; (E) Reddish-brownish watery fetid discharge. Images courtesy of Jessica Prim, Segundo Casaro, and Klibs Galvao.

A landmark study attempted to create criteria to improve uterine disease diagnosis consistency by farmers and researchers (Sheldon et al., 2006). Puerperal metritis was defined by an enlarged, flaccid uterus, a fetid, watery red-brown discharge (Figure 1E - discharge presented in a tray), concurrently or not with fever, anorexia, depression, decreased milk yield and feed intake within ten days post-calving (Sheldon et al., 2006). Endometritis was subdivided in clinical and subclinical presentations with clinical endometritis defined by the presence of purulent vaginal discharge detectable 21 days or more after parturition (Figure 1C – discharge presented in a tray), or mucopurulent discharge detectable in the vagina after 26 days postpartum. Subclinical endometritis was characterized by inflammation of the endometrium measured by the relative presence of polymorph nuclear leukocytes (PMN) in a uterine sample (collected by uterine flushing or cytobrush) in the absence of clinical disease (Sheldon et al., 2006). Considering that this article was cited 1200 times until May 31^st^ of 2020, it is fair to assume that it improved how researchers defined uterine diseases. Despite its success in the academic environment, few issues ensued since its publication. First, the term for clinical endometritis has been questioned because a large proportion of cows presenting pus in the vaginal discharge do not have concurrent neutrophil infiltration and pus in the endometrium, thus, the purulent vaginal discharge was suggested as an alternative name for clinical endometritis (Dubuc et al., 2010).

**Figure 2:**
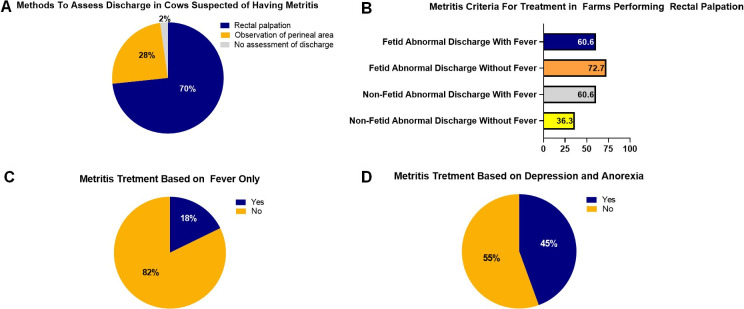
(A) Methods to assess discharge in cows suspected of having metritis; (B) metritis criteria for treatment in farms performing rectal palpation; (C) metritis treatment based on fever only; and (D) metritis treatment based on depression and anorexia.

Moreover, the term cytological endometritis has been proposed as a “universal” definition of endometritis based on the presence of PMN in the uterine lumen in cows with or without purulent vaginal discharge (Dubuc et al., 2010). Secondly, there is evidence that the criteria used to define puerperal metritis have not been used consistently in dairy farms (Espadamala et al., 2018). Researchers collected information based on cow-side observations, and responses from personnel evaluating early postpartum dairy cows revealed that 70% of the farms perform rectal palpation to retrieve vaginal discharge for metritis diagnosis (Figure 2A).

In contrast, the other 28% only observe discharge in the perineal area, and 2% perform no diagnosis of metritis (Figure 2A). Amongst the farms that performed rectal palpation, 36.3% treated cows without fetid discharge, and fever, 60.6% treated cows without fetid discharge with fever, 72.7 treated cows with a fetid discharge without fever, and 60.6% treated cows with fetid discharge and fever (Figure 2B). A total of 18% treated metritis with antibiotics based on fever only (Figure 2C), and 45% of farms treated metritis based on depression and anorexia (Figure 2D). The results of this study likely reflect the situation in many other parts of the world where the set of criteria to treat metritis is not consistent, and it may lead to over or under-diagnosis of metritis and questionable use of medically necessary antimicrobial in dairy farms (Espadamala et al., 2018; WHO, 2017).

The root of this problem is more profound than having a strict criterion that includes the presence of abnormal fetid discharge, fever, and other systemic signs such as depression and anorexia. A list of factors such as frequency of cows' examination, training of farm personnel on how to accurately identify abnormal fetid discharge, sense of smell, reporting of diagnosis and treatment, overseeing of antimicrobial use all contribute to a burden in the routine of dairy farms that leads to the inconsistencies diagnosing uterine diseases in dairy cows. Furthermore, the absence of one of the criteria, such as fever or anorexia or depression, does not eliminate the risks of culling and reduced productive and reproductive performance in cows diagnosed with metritis (Lima et al., 2014, 2019). It is reasonable to surmise that the inconsistency of diagnosing uterine diseases is a systematic problem of the dairy industry, and it will require coordinated action between farmers, researchers, dairy industry-related personnel, and government to improve judicious use of antibiotics.

### Advances in diagnosing and predicting uterine diseases

Some of the current efforts to improve the diagnosis of uterine diseases include the assessment of new technologies such as activity monitors, biomarkers, immune cell profiling, and machine learning algorithms (Barragan et al., 2018, 2019; Bogado Pascottini et al., 2020; Dervishi et al., 2018; Pomeroy et al., 2017; Stangaferro et al., 2016). These technologies have great potential to help flag high-risk animals or help predict the cure and success of antimicrobial therapy, all components that could improve the judicious use of antimicrobials and diminish the negative impact of uterine diseases. A study using an automated health-monitoring system that combines rumination and physical activity revealed a sensitivity of 55% for all cases of metritis and 78% for cases of metritis and other disorders (Stangaferro et al., 2016). A second study investigating daily activity patterns and plasma concentration of biomarkers revealed a tendency for cows with metritis spent more time lying, and had higher concentrations of substance P (a marker of pain), haptoglobin (a marker of inflammation), higher prevalence of hypocalcemia, and reduced number of circulating leukocytes and erythrocytes when compared to healthy herdmates (Barragan et al., 2018). Another study revealed that cows with metritis also had increased haptoglobin in vaginal discharge (Barragan et al., 2019). A recent study from our group attempted to identify which cows related factors at metritis diagnosis were associated with metritis cure risk (Machado et al., 2020). Among cows diagnosed with metritis that were left untreated, days in milk at metritis diagnosis were positively associated with metritis cure. At the same time, the plasmatic concentration of haptoglobin tended to be negatively related to the cure of metritis. For cows with metritis left untreated that were diagnosed after 8 days in milk or had plasma haptoglobin concentration ≤ 0.54 mg/mL, milk production, pregnancy and culling risk were comparable to healthy cows. However, performance is impaired when cows that developed metritis at ≤ 8 days in milk or had haptoglobin > 0.54 mg/mL are left untreated (Machado et al., 2020). Other exciting study investigate the metabolic fingerprints in the urine of pre-metritic and metritic cows at eight, four, and one week before metritis diagnosis (Dervishi et al., 2018). The results of this study indicated that the excretion of many amino acids, tricarboxylic acid cycle metabolites, and monosaccharides, and a combination of galactose, leucine, lysine, and pantothenate at eight weeks before parturition were potential predictive biomarkers for metritis (Dervishi et al., 2018). Researchers also characterized the profile of immune cells before parturition and discovered that cows developing metritis have an increase in CD14+ monocyte counts from 42 to 14 days/at 14 days prior to calving (Pomeroy et al., 2017). Another important player on prediction of disease and diseases impact on reproduction and other critical output is the use of machine learning algorithms that have superior predictive ability for large dataset than classic multivariable models (Bogado Pascottini et al., 2020). Our group has initiated some studies on this field, and the preliminary results suggest that in the future well-structured and tested algorithms might facilitate the decision of which cows need to be treated with antimicrobials or not. Although the results of the studies and developing tools discussed are promising, a precise, feasible, and cost-effective means to diagnose, predict metritis, or predict the cure of metritis needs further investigation.

### Advances on understanding of uterine diseases pathogenesis

The latest research has coalesced the concept that uterine diseases have a long-term effect on the uterine environment, ovarian function, and fertility in dairy cows (Bromfield et al., 2015; Horlock et al., 2020; Piersanti et al., 2019; Ribeiro, 2018; Ribeiro et al., 2016; Carvalho et al., 2019). The mechanism by which the fertility of dairy cows is affected by uterine disease remains not fully elucidated. Previous research has shown that essential steps for conception success, including fertilization of oocytes, the survival of the zygote to the morula stage, elongation of the preimplantation conceptus, and survival of the fetus to term are compromised in cows experiencing uterine diseases (Lima et al., 2013; Ribeiro, 2018; Ribeiro et al., 2016; Quintero Rodriguez et al., 2019). An animal model study revealed that intrauterine administration of LPS diminished GnRH secretion by the hypothalamus (Peter et al., 1989). The reduced LH pulsatility, which suggests that uterine disease can directly affect in hypothalamic-pituitary endocrine regulation of the reproductive axis (Peter et al., 1989). Research findings also indicate that oocyte maturation in the presence of LPS at concentrations comparable to those found within the follicle compromises the developmental competence of the oocyte, increasing germinal vesicle breakdown failure and causing abnormal spindle formation (Bromfield and Sheldon, 2011). The same study also demonstrated that maturation of the cumulus-oocyte complexes required for ovulation is also perturbed, with LPS inducing cumulus expansion in the absence of gonadotropin signaling (Bromfield and Sheldon, 2011). It is startling that even when many of these events are circumvented with the transfer of a viable, high-quality embryo on d 7 of the estrous cycle, pregnancy per embryo transfer was still 5% (40% vs. 35%) lower in cows that experienced uterine diseases (Ribeiro et al., 2016). The data from the first postpartum service indicates that lower oocyte quality and compromised uterine environment are both problems for reduced reproductive performance in dairy cows previously diagnosed with uterine diseases (Bromfield et al., 2015; Horlock et al., 2020; Ribeiro et al., 2016). A recent study induced uterine infection with *Escherichia coli* and *Trueperella pyogenes,* and three months later, changes in the transcriptome of the endometrium, oviduct, and granulosa cells were still present (Horlock et al., 2020). Another longitudinal investigation from week one to nine postpartum in cows indicate evidence for concurrent and longer-term associations between the endometrial microbiota and uterine transcriptome to support pregnancy (Moore et al., 2019). Data from human literature also suggest evidence for specific uterine microbiota or associated constituent molecules, such as polysaccharide A of the *Bacteroides fragilis* capsule link to healthy physiological function, which in turn may modulate immune cell subsets needed for implantation (Benner et al., 2018). Uterine microbiota may also be crucial in protection against uterine infections by defending their niche and competing with pathogens (Benner et al., 2018). In dairy cows, the uterine microbiome of healthy and metritic cows has been characterized and indicates distinct compositional signatures that affect the ability of cure from the disease (Galvão et al., 2019a; Jeon et al., 2018).

The pathogenesis of endometritis has also been revised (Bromfield et al., 2015; Sheldon et al., 2018, 2019), suggesting that endometrial cells and even granulosa cells are capable of mount an immune response. The immune system recognizes and eliminates microbes through a pathogen-associated molecular pattern (PAMPs). Endometrial cells and granulosa cells are equipped with a series of receptors named pathogen recognition receptor (PRR) capable of recognizing PAMPs. The toll-like receptor TLR4 is PRR that binds to PAMPs activating a molecular cascade that will lead to the production IL-1β, IL-6, and TNFα, CXCL1, CCL20, and IL-8 that are part of an effective immune response. Metabolic disturbance in calcium, non-esterified fatty acid, beta-hydroxybutyrate has been linked to higher odds of developing endometritis without a clear pathway (LeBlanc, 2012, 2020; Wathes, 2012). Recent revised literature suggests that metabolic stress hampers the inflammatory response to pathogens, and glucose and glutamine, two of the primary energy sources for cells, have their abundance reduced in postpartum dairy cows likely due to some metabolic disorders such as hypocalcemia, hyperketonemia or excessive inflammation (Sheldon, 2018). The inflammatory responses aggravate metabolic stress, with cows and tissues using glucose in more significant quantities when challenged by PAMPs (Sheldon, 2018). It was discussed that perturbing glycolysis or AMP-activated protein kinase activity decrease IL-1β, IL-6, and IL-8 in the endometrium (Sheldon, 2018). A second concept proposed by another review of literature, it is that cow's ability to defend against pathogens through tolerance and resistance is compromised in cows developing endometritis (Sheldon et al., 2019). Tolerance is the cow's immune system capability to limit the disease severity caused by a pathogen burden, whilst resistance is the ability to limit the pathogen burden and is usually the function of immunity (Sheldon et al., 2019). The reviewers argued that the increasing incidence of postpartum uterine disease might be a result of failures in tolerance to pathogens in the endometrium. Once the pathogens surmount endometrial tolerance, a swift and robust innate immune response is vital to counteract the disease-causing agent (Sheldon et al., 2019). As pointed by reviewers, metabolic stress associated with lactation compromises both tolerance and immunity, and failures in endometrial tolerance to pathogenic bacteria and the subsequent innate immune response shape postpartum uterine disease. The participation of adaptive immunity has been proposed by another review of the literature (Machado and Silva, 2020). Although a uterine protective role of adaptive immunity has not been elucidated in dairy cows, lymphoid aggregates exist in the endometrium after parturition. However, it remains elusive if development and functional profile are driven by immunization or previous exposure to pathogens. Considering the importance of innate immunity to the defense of the postpartum uterus, and the nature of most relevant uterine pathogens, the authors proposed that an adaptive immunity biased towards a Th17 response could enhance the ability of the innate immune system eliminate bacteria in the bovine endometrium after parturition (Machado and Silva, 2020). Another novel concept related to the development and progression of endometritis is differential expression of microRNAs (miR) (Jiang et al., 2020; Salilew-Wondim et al., 2016). MicroRNAs are a class of small non-coding single-stranded RNA molecules (usually 20‐22 nucleotides in length) that bind to the 3′‐untranslated regions or coding sequences of specific target mRNAs, thereby resulting in their translational repression or degradation. A study characterized the profile of miR in dairy cows revealing that miR-148 had differential expression in cows with endometritis (Salilew-Wondim et al., 2016). A follow-up study reported that miR‐148a expression in lipopolysaccharide‐stimulated endometrial epithelial cells was reduced and overexpression of miR‐148a using agomiR markedly lowered the proinflammatory cytokines, such as IL‐1β and TNF‐α (Jiang et al., 2020). Moreover, overexpression of miR‐148a also suppressed NF‐κB p65 activation by targeting the TLR4‐mediated pathway. The authors concluded that pharmacologic stabilization of miR‐148a might represent a novel therapy for endometritis (Jiang et al., 2020).

The conceptual theory about tolerance and resistance is exciting, but it needs further investigation. Hitherto, remains unclear if the issue leading to development and progression of endometritis is mainly mediated by host immune tolerance to pathogens that was disturbed by metabolic stress, adaptive immune response, miR variable expression, or factors such as the pathogens arsenal of virulence factors triggering dysregulation of the immune response.

### Advances on understanding of uterine diseases microbiota

A recent paper revised the literature of uterine disease microbiology and proposed to divide research findings into three phases (Galvão et al., 2019a). Phase one goes from the inception of research findings in the microbiology of uterine diseases until before 2010 (Galvão et al., 2019a). In phase one, studies were culture-dependent, and it had a mix of metritis and clinical endometritis-purulent vaginal discharge as the base for its analysis because the precise definition of metritis as the field knows today was only published in 2006 (Sheldon et al., 2006). The revised literature of culture-dependent studies suggested that *Trueperella pyogenes (T. pyogenes)* is a critical pathogen involved in the development of clinical endometritis and that gram-negative anaerobes such as *Fusobacterium necrophorum, Porphyromonas levii, and Prevotella melaninogenica* may act synergistically with *T. pyogenes* to cause clinical endometritis (Galvão et al., 2019a). Culture-dependent studies also endorse the contribution of *Escherichia coli* either as the primary pathogen or as a pioneer pathogen that sets up the stage for *T. pyogenes* and gram-negative anaerobes. Albeit there was only a paucity of data for cows with metritis, *T. pyogenes*, *E. coli*, and gram-negative, anaerobes seemed to be involved with the development of metritis (Galvão et al., 2019a). Regarding bacteria associated with uterine health, the revised literature of culture-based studies indicated that streptococci, or more specifically, α-hemolytic streptococci, are positively related to uterine health with some other indications that *E. coli* detected later in lactation could be related to improved uterine health. Still, these findings were inconsistent (Galvão et al., 2019a).

The second uterine disease microbiology phase initiated around 2010, when culture-independent studies used PCR techniques to investigate the microbiota of cows with metritis and clinical endometritis. During this phase, a pioneer study using multilocus sequence typing characterized specific strains of *E. coli* from animals with endometritis (Sheldon et al., 2010). These strains were more adherent and invasive to endometrial epithelial and stromal cells than *E. coli* isolated from healthy cows, and a mannose-binding assay pointed to the involvement of *fimH*. This virulence factor encodes type 1 fimbrial D-mannose specific adhesion, which is central to the pathogenicity of *E. coli* in the urogenital tract of women (Tchesnokova et al., 2011). A contemporary study screened for the presence of 32 virulence factors in *E. coli* isolated from the uterus of dairy cows within the first week postpartum and observed that prevalence of metritis was higher in cows with *E. coli* possessing *fimH* (Bicalho et al., 2010). Follow-up research findings from the same laboratory revealed that cows positive for *fimH* at one to three days postpartum were more likely to be diagnosed with metritis at eight to ten days postpartum and with endometritis at 34 to 35 days postpartum (Bicalho et al., 2012).

Furthermore, cows positive for *fimH* at one to three days postpartum had higher odds of being positive for *F. necrophorum* expressing leukotoxin A (*lktA*) at eight to ten days postpartum, which ultimately lead to increased odds of being diagnosed with metritis at eight to ten days postpartum. Additionally, cows positive for *F. necrophorum lktA* at 34 to 36 days postpartum had higher odds to be positive for *T. pyogenes fimA*, which in turn had the most significant positive correlation with clinical endometritis at 34 to 36 days postpartum (Bicalho et al., 2012). The associations of the virulence factors of the latter study were the foundation for the development of metritis vaccines discussed in the next section.

The third phase of uterine disease microbiology took place right after the 2010s, and it can be named the metagenomic phase. Albeit PCR contributed significantly to investigating specific pathogens and its virulence factors, it had limit capabilities when characterizing the entire microbial community was the goal. Hence, metagenomic sequencing was used to overcome this limitation and describe the bacterial community of cows with and without uterine diseases (Galvão et al., 2019a). A plethora of studies helped to reshape the knowledge of the uterine microbiome (Bicalho et al., 2017a, b; Cunha et al., 2018; Galvão et al., 2019b; Jeon et al., 2015, 2016, 2017, 2018; Knudsen et al., 2016; Santos et al., 2011; Sicsic et al., 2018). Some of the highlights include the assessment of the origin of the microbiome found in the uterus (Jeon et al., 2017). Throughout the years was suggested that uterine bacteria ascend from the vagina or through the vagina from the environment during parturition when the cervix is open (Sheldon and Dobson, 2004). Bacteroides, Fusobacterium, and Porphyromonas are pathogens associated with metritis that are commonly found in the rumen of cows and shed in feces. Thus, ascending uterine contamination from the environment could contribute to the development of metritis. But also, a specific uterine pathogen, Fusobacterium necrophorum, is known to gain access to the circulation when rumen acidosis occurs, and it leads to liver abscesses in cows (Tadepalli et al., 2009). Considering the scenario described above, the hematogenous contamination of the uterus with pathogens was also investigated (Jeon et al., 2017). The investigation revealed that Bacteroides, Porphyromonas, and Fusobacterium were part of the core genera in blood, feces, and vagina. However, other uterine pathogens such as Prevotella and Helcococcus were not part of the core genera in vaginal samples. Moreover, uterine pathogens showed a strong association with each other in the network of blood microbiota, but not in feces or vagina, suggesting that hematogenous uterine contamination might be necessary for the etiology of uterine diseases. 

Other highlights of Galvão et al. (2019a) literature review include bacteria present in the uterus even before calving. Indeed, cows had an established uterine microbiome within 20 minutes of parturition that did not differ between cows developing metritis and healthy cows up until two days postpartum, after which the microbial make-up of cows developing metritis deviated towards a higher mean relative of abundance Bacteroidetes and Fusobacteria and less Proteobacteria and Tenericutes (Galvão et al., 2019a). Cows with metritis have a loss of heterogeneity and a decline in microbial richness. Bacteroides, Porphyromonas, and Fusobacterium appear to behave synergistically at the genus level to cause metritis. At the species level, Bacteroides pyogenes, Porphyromonas levii, and Helcoccus ovis represented potential emerging uterine pathogens, and Fusobacterium necrophorum was confirmed as one of the species mainly associated with metritis (Galvão et al., 2019a). Failure to cure metritis was related to the higher relative abundance of Bacteroides, Porphyromonas, and Fusobacterium, and a further reduction in bacterial diversity. The authors of the revised literature proposed that metritis is linked with dysbiosis of the uterine microbiota characterized by diminished richness, and an increase in Bacteroidetes and Fusobacteria, particularly Bacteroides, Porphyromonas, and Fusobacterium (Galvão et al., 2019a).

Conversely of the second phase of research findings in uterine disease microbiology, the results of metagenomic sequencing data were not yet translated into the development of new preventatives. Considering the rapid advance and feasibility of shotgun sequencing (Hillmann et al., 2018), we will likely see more progress in uterine microbiome insights with better characterization of virulence factors for the potential new order of pathogens associated with uterine diseases. The better characterization of virulence factors and strain of specific species of bacteria can be the foundation for the development of more efficacious therapeutics to control uterine diseases.

### Alternative therapies to mitigate detrimental impacts of uterine disease in dairy cows

Motivated by the global threat of antimicrobial resistance dissemination and the World Health Organization guideline on the use of medically important antibiotics (WHO, 2017), researches embraced the endeavor of developing alternatives therapies to reduce the judicious use of antimicrobials in dairy cows due to uterine diseases. The alternative therapies claimed primarily to reduce bacterial proliferation and presence (e.g., chitosan microparticles, essential oils, mannose, bacteriophage, dextrose, and probiotics), or improve immunomodulation in the uterus (pegbovigrastim, recombinant bovine IL-8, ozone, paraffin, and vaccines). These novel therapies were described for prevention of metritis (chitosan microparticles, mannose, bacteriophage, probiotics, pegbovigrastim, recombinant bovine IL-8, and vaccines), treatment of metritis (essential oils and chitosan microparticles), treatment of clinical endometritis (dextrose and paraffin), and prevention of subclinical and clinical endometritis (ozone and dextrose).

#### Chitosan microparticles

Chitosan is a compound recognized as safe (GRAS) by the FDA, synthesized from chitin, a structural component of the exoskeleton of arthropods and the cell walls of fungi and yeast. Chitosan is a linear polysaccharide produced by the deacetylation of chitin and is nontoxic, bioadhesive, biocompatible, and biodegradable (Baldrick, 2010). This compilation of desirable traits makes chitosan a widely used component in food, pharmaceutical, textile, water treatment, cosmetics, and agriculture industries. Previous studies revealed that chitosan microparticles (CM) have broad-spectrum antimicrobial activity at acidic and neutral pH (Jeon et al., 2014), making it a promising alternative to conventional antimicrobials. A study investigated the effects of intrauterine infusion of CM on the day after calving to prevent metritis (Daetz et al., 2016). Treatment with CM decreased the incidence of metritis at seven days in milk when compared with control cows (46.2 vs. 65.4%). But differences in the rate of metritis were not present at day 4 (11.5% vs. 17.3%), 10 (61.5% vs. 73.1%), and 14 postpartum (63.5% vs. 73.1%). A recent study enrolling 826 cows with metritis from three dairies located in northern Florida investigated the effects of intrauterine infusion of 24 g of CM administered at metritis diagnosis, and day two and four after diagnosis in comparison with ceftiofur crystalline-free acid and control untreated with cows with metritis (Oliveira et al., 2020). Surprisingly, treatment with CM not only failed to improve the cure for metritis, but it also impaired milk yield, survival, and fertility when compared control untreated with cows with metritis (Oliveira et al., 2020). Therefore, the promising prospects of CM are not an option to consider for the treatment of metritis. The authors speculated that the negative impacts of CM might be at least in part due to uterine related inflammatory conditions, such as metritis, pelvic inflammation, peritonitis, and mass in the pelvis (Oliveira et al., 2020).

#### Essential oils

**Figure 3:**
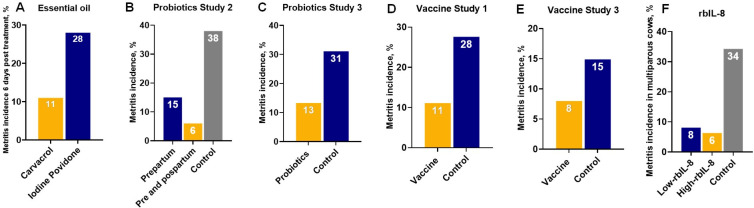
Alternative therapies for metritis prevention or treatment with positive outcomes: (A) effects of essential oil based on carvacrol (Optimum UterFlush®) on incidence of metritis six days after treatment; (B) effects of an intravaginal cocktail of lactic acid bacteria composed of Lactobacillus Sakei FUA3089, Pediococcus Acidilactici FUA3138, and Pediococcus Acidilactici FUA3140 pre (one and two weeks prepartum) and pre and postpartum (one and two weeks prepartum and one week after calving) on incidence of metritis; (C) effect of an intravaginal cocktail composed of Lactobacillus rhamnosus CECT 278, Pediococcus acidilactici CECT 5,915, and Lactobacillus reuteri DSM 20016 given twice weekly during the three-weeks pre-calving on incidence of metritis; (D) effect of vaccine composed by major virulence factors (fimH; leukotoxin, LKT; and pyolysin, PLO) inactivated whole cells of two strains of Escherichia coli, Fusobacterium necrophorum and Trueperella pyogenes administered subcutaneously at 60 and 30 days before expected calving date on incidence of metritis; (E) effect of vaccine composed recombinant subunit proteins FimH, Plo, and Lkt administered subcutaneously at 240 ± 3 and 270 ± 3 of gestation on incidence of metritis; and (F) effect of high (11.25 µg) and High (1,125 µg) of recombinant bovine interleukin-8 (rbIL-8) within 12 h of calving on incidence of metritis.

A study compared the efficacy for metritis cure of an intrauterine solution containing a certified organic essential oil (Optimum UterFlush, Van Beek Natural Science, Orange City, IA) and intrauterine use of iodine povidone in organic dairy farms (Pinedo et al., 2015). The essential oil is based on carvacrol (4-isopropyl-2-methylphenol), a monoterpenic phenol produced by aromatic plants, including oregano that has numerous bioactivities including antioxidative, anti-inflammatory, and antibacterial properties (Baser, 2008; Friedman, 2014; Suntres et al., 2015). Depletion of the intracellular ATP pool, a change in membrane potential, and an increase in the permeability of the cytoplasmic membrane for protons and potassium ions have been reported and suggested as a potential mechanism of action to diminish bacterial proliferation (Friedman, 2014). The results of the study indicated that cows treated with the essential oil based on carvacrol had a lower incidence of metritis at days six (Figure 3A) and 14 after the first treatment, and increased odds of pregnancy at the first AI, 150 days postpartum, and 300 days postpartum when compared to control cows treated with iodine povidone. Although the results are encouraging further investigation in conventional dairy farms where cows are exposed to a different environment and antimicrobials rather than iodine povidone (a product with unknown effects for uterine diseases) is warranted to see if these benefits can be replicated.

#### Mannose and bacteriophage

A study investigated the effects of intrauterine administration of mannose or a bacteriophage cocktail and the presence of *E. coli* and *T. pyogenes* in the uterine lumen on uterine diseases and reproductive performance of dairy cows (Machado et al., 2012). Mannose was investigated because the compound is an antagonist of *fimH*, the major virulence factor of *E. coli,* and higher odds of developing metritis (Bicalho et al., 2012). Bacteriophages are viruses that are obligate intracellular parasites of bacteria because they lack their metabolism. Phages are host specific, capable of infecting only particular species or even strains of bacteria. The research group developed a cocktail of bacteriophage that was capable of inhibiting the *in vitro* growth of *E. coli* isolates from metritic cows (Santos et al., 2016). Unfortunately, the results of their study revealed no effects of both mannose and the cocktail of bacteriophages on uterine health, reproduction performance, or responses in cultures for *E. coli* and *T. pyogenes* (Machado et al., 2012). Thus, mannose and bacteriophages are alternatives that will not likely become mainstream.

#### Dextrose

A series of studies investigated the use of dextrose to treat clinical endometritis in the last decade (Ahmadi et al., 2019; Brick et al., 2012; Machado et al., 2015; Maquivar et al., 2015). The results are inconsistent. Dextrose's mechanism of action to reduce clinical endometritis is unclear. Still, researchers justify its use on the ability of sugar (hypertonic sucrose) to inhibit *E. coli* growth in human wounds or how the ability of other sugar (mannose) inhibits the adhesion of *E. coli* to equine endometrial cells. After its use in cows, researchers suggested that dextrose effects could be mediated by the prevention of bacterial growth, improvement of uterine tone, and nurturing of endometrial cells. The first study evaluating the use of 200 ml of 50% dextrose intrauterine at 26 days in milk revealed a reduced incidence of clinical endometritis 14 days after treatment when compared to control cows (21.3% vs. 32.1%) and improved pregnancy per AI at the first service (29.8% vs. 21.1%). The same research group performed a second study in cows diagnosed with clinical endometritis (purulent vaginal discharge) at 26 days in milk and randomly assigned cows to receive either 200 mL of 50% dextrose intrauterine or to remain as untreated control (Maquivar et al., 2015). Once again, cows treated with dextrose had a lower incidence of clinical endometritis 14 days later (34.5% vs. 42.7%) and improved pregnancy per AI at the first service (29.8% vs. 22.7%). A third study performed from a different research group diagnosed cows with clinical endometritis at 30 ± 3 days in milk and randomly assigned cows to receive either 200 mL of 50% dextrose intrauterine or to remain as untreated control (Machado et al., 2015).

Conversely of first two studies, treatment with 200 mL of 50% dextrose intrauterine, had a robust statistical tendency to reduce the cure rate of cows with clinical endometritis, failed to improve first-service conception rate and early embryonic mortality, and did not decrease calving-to-conception interval (Machado et al., 2015). The fourth study cows were diagnosed for clinical endometritis at 30 ± 3 days and treated with one of five treatments: (1) 200 mL of 50% dextrose intrauterine; (2) intrauterine infusion of 5g of oxytetracycline; (3) PGF_2α_ – 500 mg of cloprostenol sodium; (4) 100 mL of paraffin liquid intrauterine; or (5) left untreated. Although no cure rates for endometritis were evaluated in this study, the reproductive performance of cows treated with dextrose was not improved when compared to cows left untreated (Ahmadi et al., 2019). The inconsistent results suggest that more research is needed to consolidate if dextrose is a beneficial therapy for the treatment of clinical endometritis.

### Paraffin

A study evaluated the use of liquid paraffin to treat cows with endometritis (Ahmadi et al., 2019). The rationale for liquid paraffin is the promotion of migration of macrophages and other phagocytes to improve local immune response in the uterus. In the study, cows were diagnosed for clinical endometritis at 30 ± 3 days were assigned randomly to one of five treatments: (1) 200 mL of 50% dextrose intrauterine; (2) intrauterine infusion of 5 g of oxytetracycline; (3) PGF2α – 500 mg of cloprostenol sodium; (4) 100 mL of paraffin liquid intrauterine; or (5) left untreated. The results of the study indicated that the use of intrauterine paraffin impaired reproductive performance when compared to untreated control cows (Ahmadi et al., 2019). Thus, liquid paraffin should not be considered an option to treat endometritis.

### Ozone

A study evaluated the effects of ozone on the incidence of subclinical endometritis and reproductive performance (Escandon et al., 2020). The rationale presented to use ozone it is the effects on the inhibition or inactivation of infectious microorganisms such as bacteria, fungi, spores, and viruses, and anti-inflammatory action enhanced by inhibition of proinflammatory cytokines and phospholipase A2 and by the stimulating activity of immunosuppressive cytokines, such as IL10 and TNF β1 (Sagai and Bocci, 2011). At day 35 after calving, cows were treated with 50 mL of ozonized intrauterine distilled sterile solution (50 μg/mL) or were left untreated immediately after the first endometrial cytology. The second endometrial cytology revealed that ozone treatment reduced the incidence of subclinical endometritis (5.0 vs. 50.0%) when compared to control cows. After ozone treatment, first service conception rates increased (50.0 vs. 16.2%) when compared to control cows. Although the results were very positive, the unusual meager first service conception rates and high prevalence of subclinical endometritis in control cows in an underpowered study (only 40 cows per treatment) poses the probability of type-II error in the study. Thus, further studies with larger sample size in farms with conception rates more aligned to the reality of most farms are warranted. 

### Probiotics

Three studies evaluated the effects of intravaginal infusion of a cocktail of lactic acid bacteria either on clinical endometritis/purulent vaginal discharge incidence (Ametaj et al., 2014) or metritis incidence (Deng et al., 2015; Genis et al., 2018). In the first study, the effects of a mixture of lactic acid bacteria on the incidence of purulent vaginal discharge/clinical endometritis, plasma haptoglobin concentrations, and milk production in dairy cows were investigated (Ametaj et al., 2014). The cocktail of lactic acid bacteria was composed of *Lactobacillus Sakei FUA3089, Pediococcus Acidilactici FUA3138,* and *Pediococcus Acidilactici FUA3140* and treatments were performed once a week starting two weeks before calving until week four postpartum (Ametaj et al., 2014). Intravaginal administration of the cocktail decreased the incidence of clinical endometritis at three weeks postpartum and reduced acute phase protein haptoglobin in weeks two and three postpartum. No overall effects on pregnancy rate were observed, but the treated multiparous cows produced more milk than their control counterparts (Ametaj et al., 2014). A follow-up study from the same research group evaluated the effect of the same cocktail of lactic acid bacteria on the incidence of metritis and markers of immunological status and metabolism. Treatment with the lactic acid cocktail prepartum (one and two weeks before calving) or pre and postpartum (one and two weeks before calving and at one week postpartum) reduced the incidence of metritis when compared to control untreated herdmates (Control = 38.0% vs. Only prepartum = 15.0% vs. Pre and Postpartum = 6.0%) (Figure 3B). Moreover, the authors reported a lowered incidence of total uterine infections of postpartum dairy cows associated with enhanced vaginal mucus secretory immunoglobulin A (Deng et al., 2015). Cows administered intravaginally with lactic acid bacteria had lower systemic inflammation as denoted by lower concentrations of lipopolysaccharide-binding protein and a tendency of lower serum amyloid A in the serum of the treated cows.

A third study tested the effects of a different cocktail of lactic acid bacteria administered intravaginally and intrauterine on the incidence of metritis, non-esterified fatty acids concentrations, and gene expression of proinflammatory cytokines in blood neutrophils and endometrium (Genis et al., 2018). The cocktail was composed of Lactobacillus rhamnosus CECT 278 (Colección Española de Cultivos Tipo, Valencia, Spain), Pediococcus acidilactici CECT 5,915, and Lactobacillus reuteri DSM 20016 (German collection of microorganisms and cell cultures, Leibniz, Germany) with a final cell count of 4.5 × 10^10^ CFU/dose and a proportion of 25/25/2, respectively. Treatment assignments were: (1) two intravaginal doses of cocktail weekly during the three-weeks pre-calving; (2) an intra-uterine dose, once one day after calving; and (3) control without intervention. The intravaginal treatment with lactic acid reduced incidence of metritis from 31.1% in control to 13.3% (Figure 3C). Moreover, uterine and vaginal with lactic acid treatment reduced blood neutrophil gene expression. Taken together, it seems that intravaginal probiotics are effective in reducing the incidence of metritis and endometritis. Still, its mechanism of action needs further elucidation, and more replicates in extensive field trials are required to confirm the benefits of this promising therapeutic to mitigate the negative impact of uterine diseases.

#### Metritis vaccines

In recent years, three peer-reviewed studies investigated immunizations against metritis pathogens (Freick et al., 2017; Machado et al., 2014; Meira Jr. et al., 2020). The first vaccine containing different combinations of proteins representing major virulence factors (*fimH*; *lktA*; and pyolysin, *Plo*) for uterine pathogens and/or inactivated whole cells (*Escherichia coli, Fusobacterium necrophorum,* and *Trueperella pyogenes*) administered either intravaginally or subcutaneously. Three formulations were tested comprised of virulence factors and whole cells (Formulation 1), just virulence factors (Formulation 2), and only whole cells inactivated (Formulation 3). Vaccinations were performed approximately 60 days before calving, with a follow-up booster 30 days later. The serological effectiveness of the vaccines was evaluated according to plasma levels of IgG specific to the antigens utilized in the vaccine formulations. Cows receiving subcutaneous vaccination responded well to immunization, however intravaginally vaccinated cows failed to produce a humoral response to antigens. The most promising outcome came from Formulation 1 administered subcutaneously, which reduced the incidence of metritis from 27.6 to 11.1% when compared to controls cows (Figure 3D).

The second vaccine was a herd-specific vaccine containing inactivated whole bacterial cells of *Trueperella pyogenes*, *Escherichia coli, Streptococcus uberis,* Bacteroides, and Peptostreptococcus species obtained from uterine swabs of primiparous cows diagnosed with metritis. The vaccine was prepared by IDT Biologika GmbH (Dessau-Roßlau, Germany). Late pregnant heifers were vaccinated subcutaneously six weeks before calving, followed by a second injection three weeks later. Conversely to the first study, vaccination failed to reduce the incidence of metritis (Vaccinated = 46.0% and Control = 48.9, *P* = 0.59). Clinical endometritis incidence, fertility outcomes (e.g., calving-to-conception interval, pregnancy per AI at first service), and 100-day milk production also did not differ between vaccinated and control cows.

The third study with vaccines for metritis was a follow-up to the study 1, listed above (Meira Jr. et al., 2020). It was a clinical trial that randomly assigned heifers into 1 of 4 different treatments: Control, vaccine 1 (bacterin and subunit proteins), vaccine 2 (bacterin), and vaccine 3 (recombinant subunit proteins). Heifers were immunized with a subcutaneous injection of its respective treatment at 240 ± 3 and 270 ± 3 d of gestation (Meira Jr. et al., 2020). All vaccines were prepared by Merck Animal Health (DeSoto, Kansas, USA). Vaccine 1 had two strains of inactivated utero-pathogenic *E. coli* (12714–2 and 4612–2), *T. pyogenes* (10481–8 and 6375–1), *F. necrophorum* (5663 and 513), and recombinant *fimH*, *Plo*, and *lktA* proteins blended with aluminum hydroxide. Vaccine 2 had just the inactivated *E. coli* (12714–2), *T. pyogenes* (10481–8), and *F. necrophorum* (5663) antigens blended with aluminum hydroxide. Vaccine 3 was formulated with recombinant subunit proteins *fimH*, *Plo*, and *lktA*. Control cows received a placebo containing aluminum hydroxide but no bacteria or subunit protein antigens. Immunization for metritis decreased incidence of puerperal metritis when compared with control (9.1% vs. 14.9%, respectively), and vaccine 3 alone reduced the incidence of puerperal metritis when compared with the control (8.0% vs. 14.9%, respectively) (Figure 3E). Immunization, especially with vaccine 3, improved reproductive performance (Meira Jr. et al., 2020). Vaccination against metritis pathogens decreased the total vaginal bacterial load and decreased the vaginal load of *F. necrophorum* at nine days in milk.

The vaccine development efforts presented in studies 1 and 3 (Machado et al., 2014; Meira Jr. et al., 2020) are going to become available for dairy producers soon and likely can be an instrumental tool to reduce the metritis burden and antimicrobials use. Considering, the insights of the ever-evolving field of uterine microbiome, it is reasonable to surmise that novel vaccines for metritis will be developed in the next decade to diversify the portfolio of tools to help mitigate the inherited cascade of adverse effects caused by metritis.

#### Pegbovigrastim

Pegbovigrastim is a recombinant bovine granulocyte colony-stimulating factor (G CSF). The G-CSF is an endogenous hematopoietic growth factor that stimulates the production and differentiation of neutrophils by progenitor cells in the bone marrow (Nagata, 1989). Some promising results in improving circulating neutrophils in cattle and reducing the incidence of clinical mastitis were reported (Canning et al., 2017; Ruiz et al., 2017). A follow-up study then investigated the effects of Pegbovigrastim (15 mg of pegylated recombinant bovine G-CSF) administered subcutaneously seven days before expected calving date and on the day after calving on (1) circulating blood cells; (2) the incidence of mastitis (clinical and subclinical) and uterine diseases; and (3) production and reproductive performance. The results demonstrated an increase in circulating polymorphonuclear leukocytes cells, but it did not translate into a reduced incidence of uterine diseases. Indeed, overall morbidity increased in cows treated with Pegbovigrastim, and not benefits in reproductive, and lactation performance werr observed (Zinicola et al., 2018). Since then, the commercial product (pegbovigrastim injection, Imrestor, Elanco Animal Health, Greenfield, IN) has been removed from the market in the US.

#### Recombinant IL-8

Interleukin-8 is a proinflammatory cytokine and the major chemoattractant for neutrophils. It is produced by smooth muscle, epithelial cells, endothelial cells, and cells of the innate immune system with toll-like receptors (Mitchell et al., 2003). Binding of IL-8 to its receptors CXCR1 and CXCR2 on neutrophil surface induces neutrophil activation, stimulates chemotaxis, and increases phagocytosis and killing ability (Mitchell et al., 2003). A study demonstrated that plasma IL-8 concentrations in cows without retained of fetal membranes from 15 days before calving to 15 days after calving was lower than in cows developing retained of fetal membranes (Kimura et al., 2002) indicating that IL-8 might be pivotal to attract neutrophils into the uterus for maintenance of uterine health. Dr. Rodrigo Bicalho’s laboratory developed a recombinant bovine interleukin 8 (rbIL-8), and rbIL-8 was used intrauterine within 12 hours of calving in low (L-rbIL8, 11.25 μg) and high doses (H-rbIL8, 1,125 μg) to evaluate its effects on the incidence of metritis (Zinicola et al., 2019). Treatment with L-rbIL-8 and H-rbIL8 reduced the incidence of puerperal metritis in multiparous cows when compared to control cows (Control = 34.3, L-rbIL8 = 8.11, and H-IL8 = 6.35%) (Figure 3F). Both the L-rbIL8 and H-rbIL8 groups produced significantly more milk, fat-corrected milk, and energy-corrected milk yields when compared with placebo-treated controls. Other companion studies confirmed the benefits of rbIL-8 for milk production and health of dairy cattle (Zinicola et al., 2019a, b). It is reasonable to speculate that this immunomodulator can trigger improved health and, consequently, milk production, but further research is needed to unfold rbIL-8 mechanisms improving lactation performance. Furthermore, a large field study in multiple farms with a large sample is needed to confirm if benefits to uterine health can be replicated.

### Non-therapeutics advances on uterine diseases prevention

#### Genomic enabled predictions

Few recent studies shed light on the potential of genomic enabled prediction to improve management and prevention of diseases such as metritis and endometritis. A first study attempted to validate genomic predictions for wellness traits in US Holstein cows (McNeel et al., 2017). The findings of this study suggested that health trait predictions were related to differences in phenotype for disease incidence among the worst and best groups. The disparity between the worst and best genetic groups in the incidence of reported disease was 1.1% for displaced abomasum, 1.7% for ketosis, 2.9% for retained placenta, 3.9% for lameness 7.4% for mastitis, and 10.8% for metritis. Authors suggested that the health traits of young calves and heifers can likely be incorporated effectively to predict differences in future health performance and can be a compelling opportunity to help reduce disease incidence and improve profitability in dairy operations (McNeel et al., 2017). A second study compared the accuracies of different Bayesian regression models in predicting molecular breeding values for health traits in Holstein cattle (Lopes et al., 2020). Genetic heritability for metritis (0.05) and clinical endometritis (0.04) were low. Genomic heritability for Bayesian models tested improved heritability of metritis to a range of 0.12 to 0.17 and heritability of clinical endometritis to a range of 0.11 to 0.21. The predictive ability models for metritis had an area under the curve ranging from 0.24 to 0.36, whereas the predictive ability models for clinical endometritis ranged from 0.66 to 0.69. The author concluded that high-density SNP panels could be successfully used to predict genomic breeding values of health traits in Holstein cattle. Still, the model choice will most likely depend on the genetic architecture of the attribute of interesting (Lopes et al., 2020).

Considering the rapid spread use of genotyping and the improvement of predictive models, it is logical to conjecture that genomic selection will become an integral part of the prevention of uterine diseases in the future.

## Conclusions

**Figure 4:**
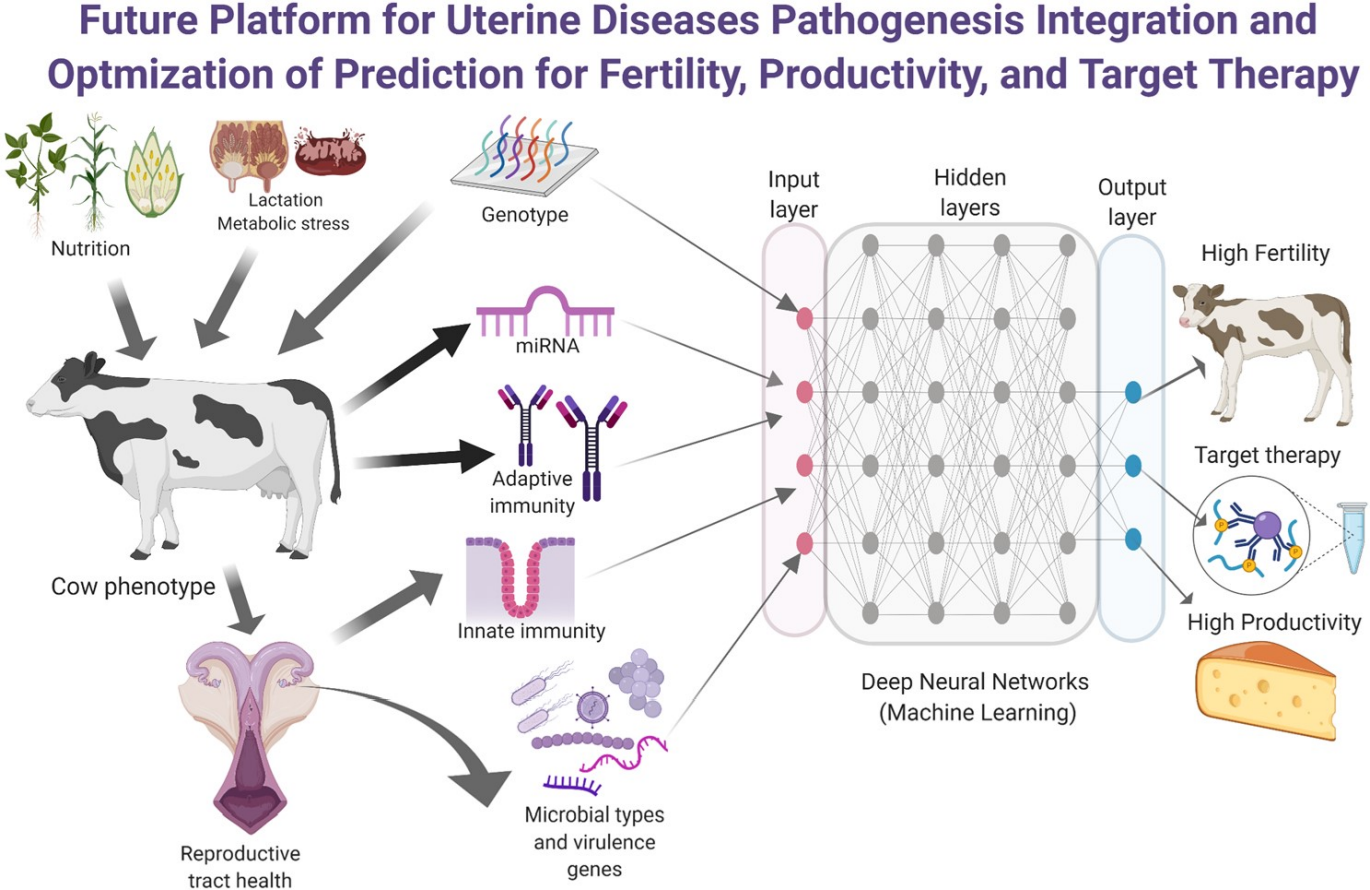
Hypothetical platform for uterine diseases pathogenesis integration and optimization of prediction for fertility, productivity, and target therapy: Current wealthy of data and its complexes interactions cannot be handled through classic statistical inference models. An optimum platform to handle large dataset could be through the use of machine learning algorithms such as deep neural networks that can significantly improve predictions for target therapies for uterine diseases, and predictive models for high fertility and productivity.

Criteria to diagnose and treat metritis and endometritis remain puzzling. Still, the incorporation of data produced from activity monitors, biomarkers, and machine learning models has the potential to refine the diagnosis and prediction of uterine diseases and its cure, leading to improved judicious use of antimicrobials. The advances in the understanding of uterine disease pathogenesis are providing insights for the development of alternative therapies to mitigate the negative impact of uterine diseases. Pivotal concepts such as the participation of adaptive immune response on uterine defense, the tolerance of cows to pathogens, participation of miR on uterine defense, need to be exploited and better understood within a systematic and structured view of cow's environmental and metabolic challenges to advance the specificity and effectiveness of novel therapies. A flurry of alternative therapies that include essential oils, vaccines for metritis, immunomodulators, and probiotics currently offer promising results but still need validation and cost-effectiveness assessment before reach mainstream for farmers use. The use of genomic enabled prediction for health traits is another advance that should soon change the management of uterine diseases in dairy farms. Despite the infancy status of many therapeutics and other technologies, it is tantalizing to conjecture that soon, many more efficacious surrogate measures to antibiotics will be available to manage uterine diseases in dairy farms. Considering the wealth of big dataset and its complexes interactions, it is rational to assume that an optimum platform to investigate the development of target therapies for uterine diseases, and predictive models for high fertility and productivity will have to be channeled through machine learning algorithms capable of handling large dataset as depicted in Figure 4.
